# An Unusual Case of Dual Left Anterior Descending Artery with Coronary Computed Tomographic Angiographic Correlation

**DOI:** 10.1155/2013/348624

**Published:** 2013-11-03

**Authors:** Kyle M. Moulton, Greg Kraushaar, Derek A. Fladeland

**Affiliations:** Department of Medical Imaging, University of Saskatchewan, 103 Hospital Drive, Saskatoon, SK, Canada S7N 0W8

## Abstract

Dual left anterior descending artery (LAD) is a rare coronary anomaly that is important to recognize at coronary imaging as it may influence reperfusion strategies. Four types of dual LAD are described by the traditional literature. We present a novel case of dual LAD with coronary computed tomographic angiographic correlation that does not fit into this classification system. Rather, our case supports the recently proposed notion of adding a fifth variant to the traditional dual LAD classification system.

## 1. Introduction

Dual left anterior descending artery (LAD) is an extremely rare coronary anomaly traditionally classified into one of four types. Recognition at coronary imaging is critical as both surgical and percutaneous reperfusion strategies may be altered by its presence [1].

## 2. Case Presentation

A 37-year-old Caucasian male presented with a 1-year history of atypical left-sided chest discomfort. Past medical history was negative for major cardiac risk factors but positive for borderline diabetes, increased body mass index, and gastroesophageal reflux. Physical exam was unremarkable. EKG demonstrated a left anterior fascicular block. Biochemical markers of cardiac ischemia were negative.

Single photon emission computed tomography stress myocardial perfusion imaging (SPECT-MPI) revealed a heterogeneous inferolateral and apical myocardial perfusion defect with partial reversibility. Conventional coronary angiography identified an aberrant left anterior descending artery (LAD) originating from the right coronary cusp without evidence of atherosclerosis. To further characterize the anatomy of this aberrant artery, coronary computed tomography angiography (CTA) was performed.

**Figure 1:**
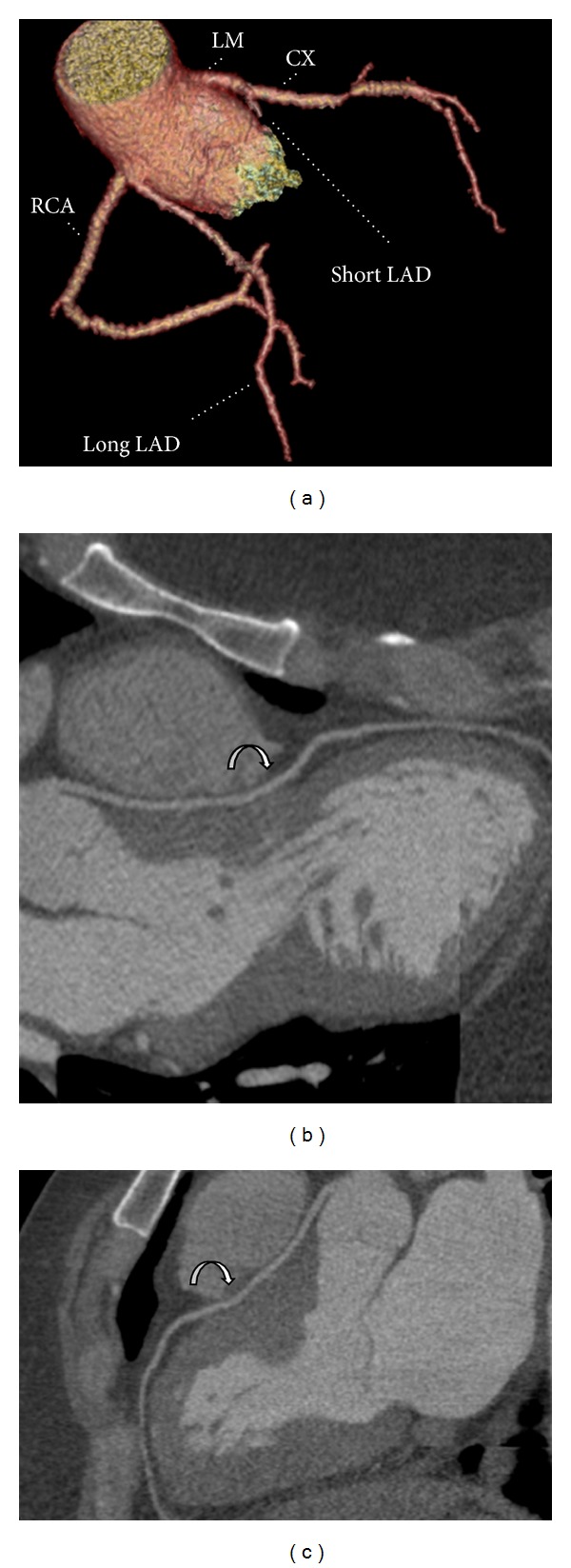
(a) 3D volume-rendered CTA images of the coronary circulation (LM: left main coronary artery; CX: circumflex branch; LAD: left anterior descending artery; RCA: right coronary artery). (b) and (c): Curved multiplanar reconstruction CTA images of the long LAD arising from the right coronary cusp with hammock-like descending curvature (curved arrows) consistent with a transseptal course.

CTA revealed a novel case of dual LAD. The long LAD segment arises from a common coronary ostium on the right coronary cusp. It takes an aberrant course posterior to the right ventricular outflow tract. The long LAD has a hammock-like downward slope that closely resembles, on coronal imaging, the hammock sign described on conventional angiography [2]. The long LAD descends inferiorly from its origin and enters the superior aspect of the interventricular (IV) septum where it provides a small septal perforator. It reemerges in the epicardium of the IV groove distal to the pulmonary trunk to give off a diagonal branch. The septal course of the aberrant long LAD presumably mitigates against the risk of sudden cardiac death associated with a malignant interarterial LAD [3]. The short LAD segment originates at the left coronary cusp, gives off a diagonal branch, and terminates high in the IV groove (Figure 1).

## 3.  Discussion

Dual LAD is a rare type of coronary artery anomaly that is important to recognize and characterize as it may critically influence surgical or angiographic reperfusion strategies [1, 4]. In dual LAD, the functional LAD is divided into a short and a long segment [1, 4]. The short LAD typically arises from the LAD proper and terminates high in the IV groove [1, 4]. The long LAD takes a more variable course around the short segment and returns to the IV groove distally [1, 4].

There are four types of dual LAD described in the traditional literature that are distinguished by the origins and courses of the long and short segments of the dual LAD system, as well as the supply of the septal perforators and diagonal branches [1, 4] (Table 1). Recently, a novel Type V dual LAD has been proposed on the basis of a single case [5]. In this variant, the long LAD originates from a common ostium on the right coronary cusp, takes an aberrant septal course, and reemerges within the IV groove to give a distal septal perforator [5]. The short LAD originates from the left coronary sinus and terminates high in the IV groove, providing a proximal septal perforator [5]. Diagonal branches were not described [5]. We propose slightly amending the Type V dual LAD to include the possibility of septal perforators arising from the long LAD only and diagonal branches arising from both the short and long LAD as has occurred in our case (Table 1). 

In summary, we present an unusual case of dual LAD that does not fit within the traditional classification system. A similar case of this anatomic variant has recently been described in the literature, which the authors have presented as a novel Type V dual LAD [5] (Table 1). Our current case supports this notion and provides further credence for adding a fifth type of variant to the traditional dual LAD classification system.

## Figures and Tables

**Table 1:** Classification of dual LAD on coronary CTA, adapted from [4].

Type	Short LAD course	Long LAD course	Origin of septal perforator	Origin of diagonal vessels
I	(i) Originates from LAD proper (ii) Terminates high in IV groove	(i) Originates from LAD proper (ii) Courses to left of IV groove (iii) Reenters distal IV groove	(i) Short LAD	(i) Long LAD (ii) LAD proper (iii) Both

II	(i) Originates from LAD proper (ii) Terminates high in IV groove	(i) Originates from LAD proper (ii) Courses to right of IV groove (iii) Reenters distal IV groove	(i) Short LAD	(i) LAD proper

III	(i) Originates from LAD proper (ii) Terminates high in IV groove	(i) Originates from LAD proper (ii) Myocardial course within IV septum proximally (iii) Epicardial course within IV groove distally or (iv) Gives terminal branches at apical IV groove	(i) Short LAD (ii) Long LAD	(i) Short LAD (ii) LAD proper

IV	(i) Originates from left main (ii) Terminates high in IV groove	(i) Originates from right coronary artery (ii) Anomalous prepulmonic course (iii) Reenters distal IV groove	(i) Short LAD	(i) Short LAD

V	(i) Originates from left coronary cusp (ii) Terminates high in IV groove	(i) Originates from right coronary cusp (ii) Aberrant septal course (iii) Reenters distal IV groove	(i) Long LAD (ii) Both	(i) Neither (ii) Both
